# The Role of Alpha-Synuclein and Tubulin-Associated Unit (Tau) Proteins in the Diagnosis, Prognosis, and Treatment of Parkinson’s Disease: A Systematic Review

**DOI:** 10.7759/cureus.64766

**Published:** 2024-07-17

**Authors:** Yi Mon Lin, Devendar Banoth, Muhammad Hassaan Wali, Khava Bekova, Noor Abdulla, Simhachalam Gurugubelli, Safeera Khan

**Affiliations:** 1 Internal Medicine, California Institute of Behavioral Neurosciences and Psychology, Fairfield, USA; 2 Surgery, California Institute of Behavioral Neurosciences and Psychology, Fairfield, USA

**Keywords:** alpha synuclein, diagnosis, parkinson’s disease, prognosis, role, tau protein, treatment

## Abstract

Parkinson's disease (PD) is a degenerative neurological disorder resulting from the death of dopaminergic neurons, which, in turn, results in impaired motor and cognitive functions. Early diagnosis is important in achieving a good prognosis for PD. Currently, the only approved way to diagnose PD is through medical history, current symptoms, and neurological examination. This, however, can only happen after PD progresses far enough in patients. Biomarkers in cerebrospinal fluid (CSF) and blood plasma, however, may provide insight into the early progress of PD and potentially concurrent dementia, which can also aid in the development of novel treatments. Specifically, this systematic review explores alpha-synuclein (α-syn) and tubulin-associated unit (Tau) proteins and analyzes their potential roles as biomarkers while also touching on nilotinib and immunotherapy as potential treatment options. PubMed, PubMed Central (PMC), Medline, and Cochrane Library serve as the databases for relevant literature, upon which eligibility criteria and quality checks - Assessment of Multiple Systematic Review (AMSTAR) tool, Newcastle-Ottawa Quality Assessment Scale, Cochrane risk-of-bias assessment 2 (RoB2), and Scale for the Assessment of Narrative Review (SANRA) - were applied. The remaining literature examines the various aspects of PD and Parkinson’s disease dementia (PDD) and associated biomarkers. From 10 studies, 3,050 participants, both PD patients and healthy controls (HCs), were assessed and compared. Various assessment scales, such as the Unified Parkinson's Disease Rating Scale part III (UPDRS III), were used to ascertain the severity or progression of PD in patients while also seeking a noticeable correlation with α-syn and total Tau (t-Tau). The lack of standardized clinical testing has led to conflicting reports. Thus, while the articles generally agree on the potential efficacy of α-syn and Tau protein analysis in the diagnosis, prognosis, and treatment of PD and PDD, they also argue for further testing and trials.

## Introduction and background

Parkinson’s disease (PD) is characterized by dopaminergic neuron death due to the build-up of alpha-synuclein (α-syn) Lewy bodies (LBs) in the brain, resulting in the deterioration of motor functions and, in approximately a quarter of patients, cognitive function as well [1]. 

Tubulin-associated unit (Tau) proteins make up the neurofibrillary tangles found inside the brain's nerve cells. Under normal conditions, they stabilize neuronal microtubules. α-syn is defined as a neuronal protein that is involved in synaptic trafficking, which precedes neurotransmitter release. It is commonly believed that its protofibrils disrupt normal cell function and can lead to harmful effects on nearby cells. An imbalance of either is suspected to contribute to cognitive impairment or gait disturbances [2]. 

Studies suggest that Tau protein and α-syn can be used as biomarkers to ascertain a PD diagnosis [1,2]. However, these studies have yielded inconsistent and sometimes even contradictory results. For example, some have shown a decrease in α-syn, whereas others showed an increase, while most exhibited further decline in cognitive and motor functions due to these changes [3].

How early a PD diagnosis is made may have a strong influence on diagnosis and prognosis [4]. Since PD shares many of its symptoms with other neurodegenerative diseases, misdiagnosis is common. Positron emission tomography (PET) and single-photon emission computed tomography (SPECT) are common imaging techniques used to diagnose PD; however, their reliance on detecting changes in dopaminergic nerve terminal density in the basal ganglia leaves them susceptible to misdiagnosis, since other diseases also share these changes [3,5]. Lumbar puncture is another method used to diagnose PD, due to cerebrospinal fluid (CSF) being close to the central nervous system (CNS) [2,3,5,6]. However, its invasiveness and potential for deleterious effects make it an unpopular procedure [2,5]. While biomarkers for PD, such as α-syn, can also appear in blood and saliva, neither is effective, as saliva has low concentrations of protein and the blood-brain barrier causes low concentrations of biomarkers related to PD in blood.

The study of biomarkers is also essential in developing potential novel treatments for PD. One such treatment is nilotinib hydrochloride, which can induce autophagic degradation of α-syn and Tau proteins, thereby reducing their concentrations [7]. Alternatively, immunotherapy has the potential to treat PD by using antibodies that specifically target α-syn [8].

This systematic review will examine the role of α-syn and Tau proteins in the diagnosis, prognosis, and treatment of PD. Additionally, the efficacy of nilotinib and immunotherapy as potential treatment options will be studied.

## Review

Methodology

This systematic review was conducted using the Preferred Reporting Items for Systematic Review and Meta-Analysis (PRISMA) 2020 guidelines [9].

Information Sources and Search Strategy

PubMed, PubMed Central (PMC), Medline, and Cochrane Library were searched for relevant literature. Various combinations of the keywords role, diagnosis, treatment, Tau protein, α-syn, and PD were used in addition to the booleans AND and OR. Table 1 displays the databases used, the respective search queries, and the identified numbers of papers for each database.

**Table 1:** Search strategy and keywords used and the resulting number of papers.

Search strategy	Database used	Number of results
(("tau proteins"[MeSH Terms]. AND "tau protein"[All Fields]. AND ("alphα-synuclein"[MeSH Terms]. AND "alphα-synuclein"[All Fields]. AND ("parkinson disease"[MeSH Terms]. AND "parkinson disease"[All Fields].)) AND (medline[sb]. AND "2013/04/25"[PDat]. : "2023/04/22"[PDat].)	PMC/Medline	61
tau protein AND alpha synuclein in parkinson disease	PubMed	234
(("tau proteins/adverse effects"[MeSH Terms]. OR "tau proteins/antagonists and inhibitors"[MeSH Terms]. OR "tau proteins/blood"[MeSH Terms]. OR "tau proteins/cerebrospinal fluid"[MeSH Terms]. OR "tau proteins/drug effects"[MeSH Terms]. OR "tau proteins/genetics"[MeSH Terms]. OR "tau proteins/therapeutic use"[MeSH Terms]. OR "tau proteins/toxicity"[MeSH Terms].) AND ("alpha synuclein/adverse effects"[MeSH Terms]. OR "alpha synuclein/antagonists and inhibitors"[MeSH Terms]. OR "alpha synuclein/blood"[MeSH Terms]. OR "alpha synuclein/cerebrospinal fluid"[MeSH Terms]. OR "alpha synuclein/drug effects"[MeSH Terms]. OR "alpha synuclein/genetics"[MeSH Terms]. OR "alpha synuclein/therapeutic use"[MeSH Terms]. OR "alpha synuclein/toxicity"[MeSH Terms].) AND ("parkinson disease/blood"[MeSH Terms]. OR "parkinson disease/cerebrospinal fluid"[MeSH Terms]. OR "parkinson disease/diagnosis"[MeSH Terms]. OR "parkinson disease/drug therapy"[MeSH Terms]. OR "parkinson disease/genetics"[MeSH Terms]. OR "parkinson disease/therapy"[MeSH Terms].)) AND ((y_10[Filter].) AND (fft[Filter].) AND (humans[Filter].) AND (english[Filter].))	PubMed MeSH	69
tau protein AND alpha synuclein in parkinson disease	Cochrane Library	10
Total		374
Total after removing duplicates		318

Eligibility Criteria

Literature was restricted to articles written in English, with free full text, and published within 10 years of 2023. Articles written in languages other than English, published more than 10 years before 2023, or not containing full text were excluded. Additionally, gray literature, proposals, and articles pertaining to genetic studies were excluded.

Selection Process

The shortlist was transferred to EndNote (Clarivate, Philadelphia, USA), then to Google Sheets (Google, Inc., Mountain View, USA), where duplicates were removed. The titles and abstracts of each article were further screened and independently assessed by Yi Mon Lin and Devendar Banoth (first and second authors). Concerns about the aforementioned eligibility criteria were discussed with all other authors and finalized by mutual consensus. The penultimate shortlist was evaluated through analysis of full texts to remove articles without sufficient contribution.

The penultimate shortlist was further screened through the use of each article’s respective quality assessment tool. Systematic reviews were assessed with Assessment of Multiple Systematic Review (AMSTAR) [10], cohort and cross-sectional studies were assessed with the Newcastle-Ottawa Quality Assessment Scale [11,12], randomized controlled trials (RCTs) were assessed with Cochrane risk-of-bias assessment 2 (RoB2) [13], and narrative reviews were assessed with Scale for the Assessment of Narrative Review (SANRA) [14]. Table 2 details the quality appraisal for cohort and cross-sectional studies.

**Table 2:** The Newcastle-Ottawa Quality Assessment Scale was used to assess the eligibility of cohort and cross-sectional studies.

Author and year of publication	Type of the study	Selection (maximum 4 stars)	Comparability (maximum 2 stars)	Outcome (maximum 3 stars)
Baek et al. 2021 [3]	Cohort study	****	**	***
Hall et al. 2015 [5]	Cohort study	****	*	***
Kang et al. 2013 [4]	Cross-sectional study	****	*	***
Myers et al. 2022 [2]	Cohort study	****	*	**
Skogseth et al. 2015 [15]	Cohort study	****	**	*
Hall et al. 2016 [6]	Cohort study	****	**	***

Table 3 details the quality appraisal for RCTs.

**Table 3:** The Cochrane Risk of Bias Assessment Tool was used to assess the eligibility of clinical trials and randomized controlled trials.

Author and year of publication	Type of the study	Was the allocation sequence random?	Was the allocation sequence concealed until participants were enrolled and assigned to interventions?	Did baseline differences between intervention groups suggest a problem with the randomization process?	Were data for this outcome available for all, or nearly all, participants randomized?
Pagan et al. 2020 [16]	Randomized controlled trial	Yes	Yes	Yes	Yes
Pagan et al. 2016 [7]	Randomized controlled trial	Yes	Yes	Yes	Yes

Table 4 details the quality appraisal for the systematic review.

**Table 4:** Assessment of Multiple Systematic Review was used to assess the eligibility of systematic reviews. PICOs: Patient/population, intervention, comparison, and outcomes; RoB: Risk of bias

Author and year of publication	Type of the study	Did the research questions and inclusion criteria for the review include the components of PICO?	Did the report of the review contain an explicit statement that the review methods were established prior to the conduct of the review and did the report justify any significant deviations from the protocol?	Did the review authors explain their selection of the study designs for inclusion in the review?	Did the review authors use a comprehensive literature search strategy?	Did the review authors perform study selection in duplicate?	Did the review authors perform data extraction in duplicate?	Did the review authors provide a list of excluded studies and justify the exclusions?	Did the review authors describe the included studies in adequate detail?	Did the review authors use a satisfactory technique for assessing the RoB in individual studies that were included in the review?	Did the review authors report on the sources of funding for the studies included in the review?	If meta-analysis was performed, did the review authors use appropriate methods for statistical combination of results?	If meta-analysis was performed, did the review authors assess the potential impact of RoB in individual studies on the results of the meta-analysis or other evidence synthesis?	Did the review authors account for RoB in primary studies when interpreting/discussing the results of the review?	Did the review authors provide a satisfactory explanation for, and discussion of, any heterogeneity observed in the results of the review?	If they performed quantitative synthesis did the review authors carry out an adequate investigation of publication bias (small study bias) and discuss its likely impact on the results of the review?	Did the review authors report any potential sources of conflict of interest, including any funding they received for conducting the review?
Leaver and Poston 2015 [1]	Systematic review	Yes	Partial yes	Yes	Partial yes	Yes	Yes	Yes	Partial yes	Partial yes	Yes	N/A	N/A	Yes	Yes	Yes	Yes

Table 5 details the quality appraisal for the narrative review.

**Table 5:** Scale for the Assessment of Narrative Review (maximum two stars each) was used to assess the eligibility of narrative reviews. RCTs: Randomized controlled trials

Author and year of publication	Type of the study	Justification of the article’s importance for the readership	Statement of concrete aims or formulation of questions	Description of the literature search	Referencing	Scientific reasoning (e.g., incorporation of appropriate evidence, such as RCTs in clinical medicine)	Appropriate presentation of data (e.g., absolute vs relative risk; effect sizes without confidence intervals)
Wang et al. 2019 [8]	Narrative review	**	**	-	**	**	**

Data Collection

Following the finalization of the shortlist, the outcomes, methodologies, and statistics were independently extracted and reviewed by Yi Mon Lin and Devendar Banoth. All co-authors then assessed the data, in addition to all other necessary information, and a common consensus was established to finalize the relevant data.

Results

Study Identification and Selection

**Figure 1:**
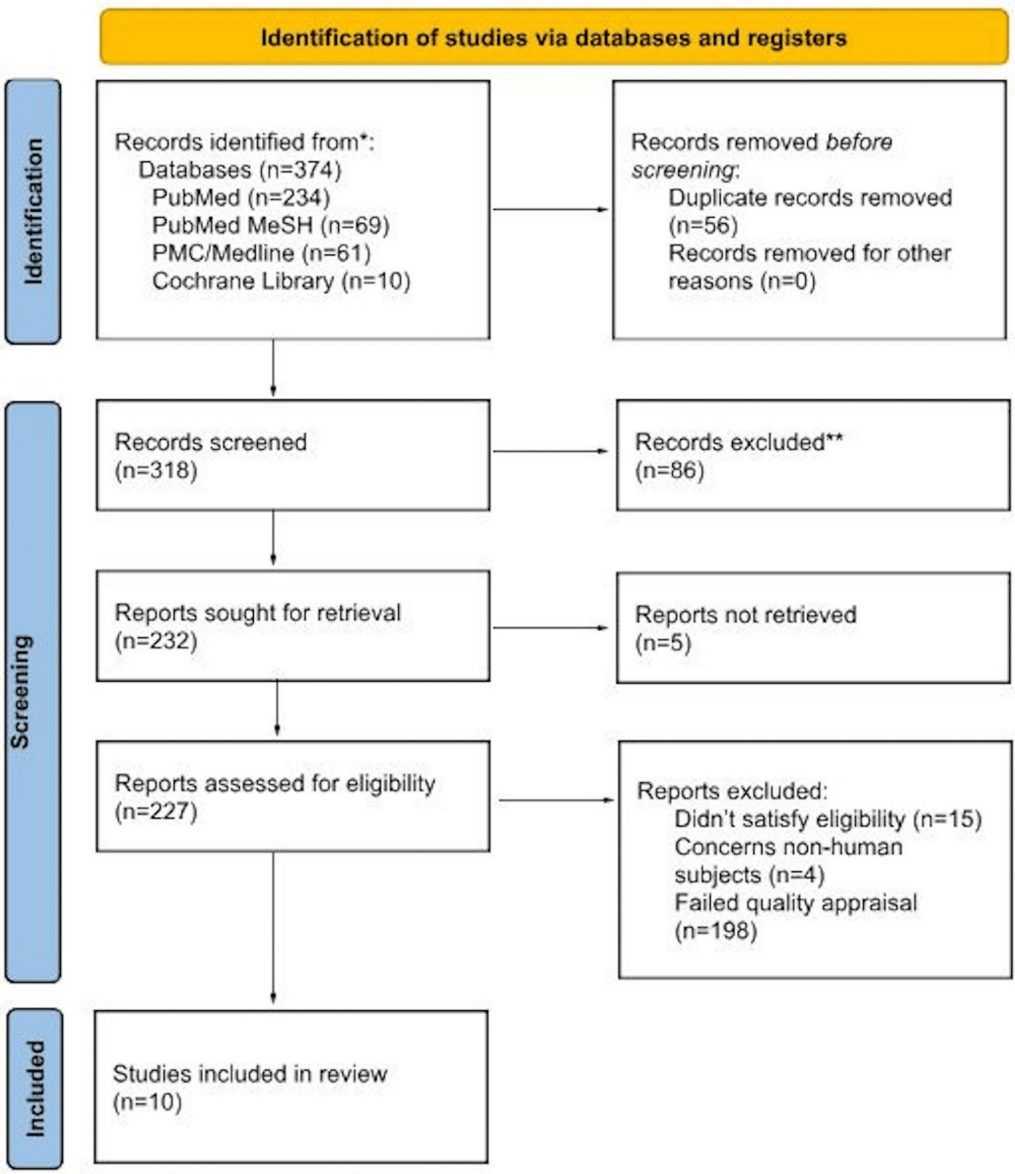
Article selection using the Preferred Reporting Items for Systematic Review and Meta-Analysis 2020 flowchart. * Databases include PubMed, PubMed MeSH, PMC/Medline, and Cochrane Library. ** Records excluded due to irrelevance, lack of necessary data, or failure to meet quality standards.

After filtering the databases through the eligibility criteria, 374 articles remained after which 56 were determined to be duplicates and, thus, removed. Further screening was conducted through evaluation of the titles and abstracts of the remaining 318 records from which 86 were excluded, five weren't retrieved, 15 failed eligibility, four concerned non-human subjects, and 198 failed quality appraisal. Ultimately, the final list consists of 10 articles which were assessed and accepted by the author and coauthors. Figure 1 shows the PRISMA 2020 flowchart used for article selection.

Outcomes Measured

The roles of Tau and α-syn in the diagnosis and prognosis of PD serve as the primary outcomes of this study. As such, the proteins’ roles in developing treatments for PD and Parkinson’s disease dementia (PDD) are secondary outcomes.

Study Characteristics

Out of the 10 articles reviewed, a total of 3,050 patients were evaluated and analyzed. The articles consist of five cohort studies, one cross-sectional study, one systematic review, one narrative review, and two RCTs. Table 6 summarizes the characteristics of each article.

**Table 6:** Details of included studies. Aβ: Amyloid-β; Aβ1-42: Amyloid-β 1-42; α-syn: Alpha-synuclein; AD: Alzheimer's disease; AQT: A quick test of cognitive speed; CSF: Cerebrospinal fluid; HC: Healthy control; LB: Lewy body; NfL: Neurofilament light; PD: Parkinson's disease; PDD: Parkinson's disease dementia; PET: Positron emission tomography; Tau: Tubulin-associated unit; p-Tau: Phosphorylated Tau; t-tau: Total Tau; TUG: Timed up and go; UPDRS III: Unified Parkinson's disease rating scale part III; YKL-40: Chitinase-3-like protein 1 (CHI3L1)

Author and year of publication	Type of the study	Purpose of the study and biomarker studied	Number of participants	Results	Conclusions
Baek et al. 2021 [3]	Cohort study	Predicting long-term temporal effects of CSF α-syn, Aβ, t-Tau, p-Tau, and serum NfL by observing and integrating the function between baseline levels and annual changes.	578	Concentrations of α-syn decreased exponentially while t-Tau and p-Tau increased prior to the onset of motor symptoms. The rates of change were higher in those diagnosed with cognitive impairment.	Amyloid burden and cognitive impairment may correlate with changes in biomarker levels. The rapid growth of LB- and AD-type pathologies may feature early cognitive decline. Vulnerability to LB pathologies, neuroaxonal damage, and cognitive impairment was apparent in PD patients with low Aβ1-42. Amyloid-lowering therapy may be useful in delaying pathological progression and cognitive decline in PD.
Hall et al. 2016 [6]	Cohort study	Investigating the use of CSF biomarkers to predict the progression of motor and cognitive decline in patients with PD	111	Over two years, increased levels of α-syn and p-Tau were linked to motor function and cognitive decline as tested with Hoehn and Yahr, UPDRS III, TUG, and AQT.	Over the course of 2 years, evidence indicates an association of cognitive and motor decline with increased levels of α-syn. It's believed that α-syn might act as a biomarker for more intense synaptic degeneration in those with PD.
Hall et al. 2015 [5]	Cohort study	Investigating the changes of CSF Tau protein and α-syn correlate with the progression of cognitive and motor decline in PD.	84	A significant correlation between concentrations of α-syn and Tau in CSF was observed. Levels of α-syn, t-Tau, and p-Tau increased over two years in the PD group whereas there were no observable changes in the HCs. Those with long disease duration exhibited increases in α-syn and Tau levels. The faster motor decline is linked to an increase in p-Tau over two years.	Over the course of two years, CSF levels of α-syn, Tau proteins, NfL, and YKL-40 increased in patients with PD. α-syn and Tau levels, in particular, remain stable in the early phases of PD but increase as the disease progresses. It is suspected that α-syn levels will increase further with neurodegeneration over time.
Kang et al. 2013 [4]	Cross-sectional study	Observing and analyzing the baseline characteristics and relationship to the clinical uses of CSF Tau proteins and α-syn as biomarkers in HCs and drug-naive patients in the early stages of PD.	102	In comparison to HCs, those with PD showed decreased levels of t-Tau, p-Tau, and α-syn. There was a significant correlation between α-syn and Tau levels.	Aβ1-42, t-Tau, p-Tau181, and α-syn levels in CSF can be used to identify early stages of PD when compared to HCs while also reflecting the various clinical features of PD.
Leaver and Poston 2015 [1]	Systematic review	Analyzing the use of CSF protein as biomarkers for prediction of cognitive impairment.	1218	There is limited evidence of the usefulness of Tau protein as a biomarker for predicting PD. Concentrations of α-syn have better potential, however, evidence for the protein as a predictive biomarker is mixed.	Biomarkers in CSF are only one of many under consideration in the diagnosis and prognosis of PDD. Used in combination with genetics, brain network connectivity, and biochemical changes, CSF biomarkers have the potential to predict cognitive decline in PD patients. When also considering demographic and clinical risk, those with the highest risk of PDD can be identified early.
Myers et al. 2022 [2]	Cohort study	Examining the predictive qualities of certain proteins in regards to longitudinal cognitive decline in PD.	204	As predictive biomarkers, α-syn and Tau served poorly while others such as amyloid-β were more effective.	PET scans of CSF levels of B-amyloid appear predictive of cognitive decline. CSF levels of α-syn and Tau protein seemed to indicate cognitive decline.
Pagan et al. 2020 [16]	Randomized controlled trial	Analyzing the effects of nilotinib in exploratory biomarkers in patients with PD.	75	The 150-mg group exhibited decreases in α-syn oligomers and p-Tau concentrations. The 300-mg group showed only a decrease in α-syn levels.	Trials of treatment with nilotinib yielded feasibly safe results. Nilotinib was detectable in CSF and exploratory biomarkers showed a response.
Pagan et al. 2016 [7]	Randomized controlled trial	Assessing the safety and feasibility of nilotinib in the treatment of PD.	75	Doses of 150 mg and 300 mg of nilotinib per day have shown minimal serious adverse effects potentially providing a safe treatment to those with advanced PD.	Collectively, observations indicate the potential of nilotinib as a potential treatment against PDD which warrants further study with larger, randomized, double-blind, placebo-controlled trials.
Skogseth et al. 2015 [15]	Cohort study	Examining the link between CSF biomarkers and PDD and comparing the differences in biomarker concentrations in CSF between patients with PDD and those with only PD	603	Reduced performance on the executive-attention domain and the composite cognition factor in the whole PD group has been linked to reduced concentrations of α-syn.	α-syn pathology is suspected to contribute to cognitive impairment in PD due to an association of concentrations of the protein in CSF with cognition.
Wang et al. 2019 [8]	Narrative review	Studying the pathological states of α-syn and analyzing the antibodies that recognize monomers or oligomers of α-syn.		Antibodies can be used to mark specific posttranslational modifications of α-syn for termination which, due to the protein's contribution towards Tau hyperphosphorylation, may also reduce concentrations of p-Tau.	Antibodies targeting α-syn have the potential to treat PD, however since α-syn is diverse, no antibody can target all post-translational modifications of the protein. Additionally, immunotherapy trials have, thus far, been restricted to the laboratory. Nonetheless, recent interest has sparked studies into more targeted treatments.

Discussion

This systematic review examines the potential of using α-syn and Tau protein as biomarkers in diagnosing and predicting prognosis in PD and the associated cognitive decline, via their concentrations in CSF and blood plasma. Additionally, studies of potential treatments targeting α-syn and total Tau (t-Tau) for PD are covered.

Pathophysiology of α-syn and Tau Protein in PD Diagnosis, Prognosis, and Treatment

Abundantly found in the brain, α-syn is a protein that transforms into LBs - aggregate and less soluble forms of the protein - in PD patients. Its function is not clearly understood, nor is the mechanism by which its levels change in PD [15].

Within the neurons of the CNS, Tau proteins are protein isoforms whose role is to maintain microtubule stability in axons. They are often associated with PD, as hyperphosphorylation of the protein converts them into neurofibrillary tangles (phosphorylated Tau, or p-Tau).

One study surmises that a lower concentration of t-Tau protein levels in CSF may be due to the protein’s interaction with other types of proteins, including α-syn. This can lead to an impediment to the release of Tau proteins in CSF [4].

Increases in t-Tau and p-Tau correspond to an increase in Tau burden on the brain. This suggests that an early appearance of LB load and an increase in the concentration of Tau proteins can result in cognitive impairment in patients with PD [3].

In patients with PD, improved dopamine metabolism was noticeably linked to decreased concentrations of oligomeric α-syn and p-Tau. Additionally, a correlation between reduced t-Tau and enhanced astrocyte activity and neurotransmitter balance was also observed. Studies have shown that nilotinib and targeted immunotherapy can reduce concentrations of α-syn and Tau, which may prove useful in treating PD [7,13].

Role of α-syn and Tau Protein in PD Diagnosis and Prognosis

In a longitudinal study conducted by Hall et al., 63 nondemented PD patients and 21 healthy controls (HCs) were tested with lumbar puncture at baseline and again during a two-year follow-up. Out of the 63 non-demented PD patients, 37 had short-disease duration PD (≤5 years), and 26 had long-disease duration (>5 years). Using numerous assessment scales, including the Unified Parkinson's Disease Rating Scale part III (UPDRS III), the Hoehn and Yahr Scale, and the Timed Up and Go test (TUG), a nurse and physician experienced in movement disorders examined PD patients to assess motor function. Cognitive function was assessed using the Mini-Mental State Examination (MMSE), the Alzheimer's Disease Assessment Scale (ADAS) items 1 to 3, and the one-minute phonetic verbal fluency test or letter fluency test. A significant correlation between α-syn and Tau levels was observed in the PD and control groups when considering patient age. In comparison, HCs showed no major changes. During the two-year follow-up, CSF Tau, p-Tau, and α-syn levels significantly increased in the long-disease duration group, whereas the levels in the short-disease duration group didn’t change significantly [5].

At baseline, the PD group had lower levels of t-Tau, p-Tau, and α-syn when compared to HCs, but in the follow-up, they yielded increased levels. This coincides with previous studies that exhibited a positive correlation between CSF α-syn and Tau proteins. Hall et al. surmise that interneuronal accumulation causes a decrease in CSF α-syn in the early stages of PD, which may also cause neuronal damage as levels increase during disease progression [5].

While α-syn and Tau protein levels remain fairly stable early on, as the disease progresses, concentrations of these proteins increase rapidly. As p-Tau levels rapidly increased, motor and cognitive functions declined rapidly when evaluated, respectively, with UPDRS III and letter fluency [5]. It is believed that α-syn levels have two modes, with levels decreasing in the early stages of PD, followed by increases, which result in neuron damage [5].

Faster motor function degeneration has been linked to p-Tau levels at baseline, along with its rate of increase. This supports the role of Tau pathology in PD. It is suggested that diseases of increased severity in the future may lead to an increase in Tau and α-syn, which may have a positive feedback effect by increasing the phosphorylation of Tau, leading to disease progression [5].

Some have argued, however, that, due to the variability in longitudinal changes in biomarker levels in CSF between patients, the usefulness of t-Tau, p-Tau, and α-syn in diagnosing and prognosing PD may be diminished. It has been suggested that, to account for this variability, in addition to other potential confounders, a larger cohort must be studied for at least two years [5].

Due to the relatively low reduction of CSF α-syn levels in most synucleinopathies, the effects of CSF α-syn concentrations have not yet been ascertained. However, CSF α-syn is noted to strongly correlate with CSF Tau, the levels of which have been determined to be markers for neurodegeneration. Recent studies have possibly indicated that more severe neurodegeneration and poor prognosis in PD are associated with high levels of presynaptic CSF α-syn, though the reasons why CSF α-syn levels and disease duration do not appear to be linked haven’t been ascertained [5].

Using the Parkinson’s Progression Markers Initiative (PPMI), Baek et al. analyzed data from 396 PD patients and 182 HCs and made long-term predictions of concentrations of α-syn, t-Tau, p-Tau, and other biomarkers in CSF, predicated on annual changes when compared to baseline, at which, in agreement with Hall et al., lower levels of t-Tau, p-Tau, and exponentially lower levels of α-syn were observed in the PD group when compared to HCs. While levels of t-Tau and p-Tau increased, α-syn decreased significantly before the onset of symptoms of motor function decline, which conflicts with the results discovered by Hall et al. [3,5].

Some longitudinal studies have shown inconsistent results regarding changes in CSF biomarkers in PD patients. Baek et al. found that using Deprenyl and Tocopherol Antioxidative Therapy for Parkinsonism (DATATOP) yielded results showing 90% of PD patients with an increase in CSF α-syn, whereas another study showed the contrary when examining the full dataset of the same cohort. In another study, CSF α-syn increased only in patients without PD after five years. The inconsistency is believed to be caused, in part, by the small amount of changes over long durations and the heterogeneity of the PD patients. More consistent results are expected with a larger and more homogeneous sample size and a study over a longer duration [3].

In a longitudinal study conducted by Myers et al., participants consisting of 152 PD patients and 52 HCs had lumbar punctures analyzing Tau proteins and α-syn. Each participant was also assessed for cognitive performance, including executive function, visuospatial function, memory, attention, and the Clinical Dementia Rating Scale Sum of Boxes (CDR-SOB). Observations showed that α-syn and Tau proteins had p-values far over 0.05 for these factors. This suggests that α-syn and Tau levels in CSF do not reflect the total levels of the protein in the brain, or at least in regions related to cognitive function. Unfortunately, using α-syn levels in CSF to diagnose PDD is challenging due to the unknown strength of correlation between total CSF α-syn and the accumulation of α-syn in the brain. This also poses the question of whether concentrations of the protein in the brain are linked to parts of the brain that come into contact with CSF [2].

In a large, multicenter cohort study conducted by Skogseth et al., 414 early and untreated PD patients and 189 HCs were assessed using multiple linear regression models with a composite cognition factor, or memory, or visuospatial, or executive-attention domains as dependent variables. Additionally, predictors were biomarker concentrations in CSF, demographic characteristics, and UPDRS III. Ultimately, Skogseth et al. found that lower levels of α-syn were linked to reduced executive-attention domain and composite cognition factor performance across the PD group, which suggests that α-syn pathology may contribute to cognitive decline in early PD patients. However, Skogseth et al. also recommend further longitudinal analysis to determine the long-term potential of using these biomarkers as predictors of future cognitive decline. To better understand the link between CSF biomarkers and cognitive and motor functions, it is essential to standardize factors, both pre-analytical and analytical, when conducting future studies [8,15].

In contrast to other studies, a systematic review by Leaver and Poston found conflicting reports on the effects of t-Tau, p-Tau, and α-syn in cognitive decline. Accordingly, none of the studies reviewed showed a significant link between levels of t-Tau at baseline and cognitive impairment during follow-ups. On the other hand, of all the articles reviewed, one showed a significant correlation between p-Tau and cognitive decline upon follow-up. Additionally, Leaver and Poston found that two articles agreed that higher levels of α-syn were linked to worsening cognitive decline, with one measuring A Quick Test of Cognitive Speed (AQT) during a two-year follow-up and the other finding cognitive function preserved, as measured by the Selective Reminding Test-Total and Delayed New Dot Test [1].

While there is strong evidence reinforcing the use of concentrations of CSF Tau as a biomarker for PD, with Tau tangles appearing in 50% of patients, in addition to being a predictor for mild cognitive impairment (MCI), there is little evidence indicating its use in predicting future cases of cognitive impairment in PD. This may allow future research to focus its resources on studying other CSF biomarkers when evaluating early PD patients [1].

While some cross-sectional studies have shown that biomarkers have been linked to cognitive decline, other longitudinal studies have yielded mixed results for most candidates. Only a small number of studies have delved into concentrations of other biomarkers, such as α-syn, neurofilament light (NfL) polypeptide, and heart-type fatty acid binding protein, of which there is some indication, albeit inconsistent, that they may help predict potential cognitive decline. These inconsistencies may be due to laboratory methodology, cohort characteristics, and outcome variability, among others [1].

It is important to consider that CSF biomarkers are only one method of assessing PD. Other biomarkers, such as genetics, brain network connectivity, and biochemical changes, contribute to cognitive decline in PD. By combining these biomarkers, among others, such as gene identification, blood markers, and magnetic resonance imaging or PET, more accurate diagnoses and prognoses can be assessed. Other factors to consider are demographic and clinical risks to identify those most vulnerable to PDD. These have the benefit of allowing early diagnoses and, subsequently, treatment [1].

A longitudinal study by Hall et al. found that, over two years, cognitive processing speed and motor symptoms appeared to worsen in PD patients with high levels of baseline α-syn, which was further corroborated by a separate study using the DATATOP cohort. The latter, however, was unable to identify any correlation between the protein and motor function decline. Similarly, motor function decline has been associated with higher levels of p-Tau. The study hypothesizes that α-syn can be released into the CSF and interstitial fluid due to increased synaptic degeneration [6].

There have, however, been contrasting results in other studies, which may be explained by differences in study methodology. For example, Hall et al. note that a study by Parnetti et al. was unable to determine if α-syn levels reflected potential cognitive decline using MMSE and Montreal Cognitive Assessment (MoCA). This differs from the study done by Hall et al., which assessed cognitive processing speed. It found that scores using AQT correlated with baseline α-syn levels in PD patients [6].

Hall et al. found that motor function decline was also linked to p-Tau levels in CSF. Genome studies have shown a link between the Tau-encoding and α-syn-encoding genes and PD. In particular, risk alleles associated with PD have been found in the former. Evidence seems to indicate molecular interactions between Tau and α-syn. As such, Tau proteins can be found in α-syn-dominant LBs in the brain, and α-syn fibrils are thought to cause the accumulation of Tau proteins through cross-seeding. This is in addition to α-syn possibly triggering Tau phosphorylation and, therefore, increased p-Tau concentrations in the striatum in PD patients. Therefore, it is suspected that α-syn and p-Tau levels are strongly linked, based on previous studies that show a connection between Tau pathology and PD synucleinopathy. It is possible that patients with more aggressive cases of PD also have higher levels of p-Tau, which, as mentioned earlier, can also exacerbate phosphorylation of Tau, thereby also exacerbating PD in a positive feedback loop. It is known that increased CSF p-Tau is characteristic of cognitive decline in Alzheimer’s disease (AD), but it is also known to be linked to motor function decline in PD. This implies distinct pathogenic pathways in PD and AD [6].

Kang et al. conducted a cross-sectional study examining 102 participants, consisting of 63 PD patients and 39 HCs from the PPMI. Using the INNO-BIA AlzBio3 immunoassay, CSF t-Tau and p-Tau were measured, whereas α-syn was measured via enzyme-linked immunosorbent assay. Diagnosis, motor, neuropsychiatric, and cognitive performance were assessed using the PPMI study protocol. Kang et al. observed lower concentrations of CSF t-Tau, p-Tau, and α-syn in PD patients when compared to HCs; however, a noticeable overlap existed between both groups. PD diagnosis was noted to be linked to lower levels of p-Tau, and the severity of motor function decline has been linked to decreased t-Tau and α-syn in CSF [4].

Emerging Therapies for α-syn and Tau Protein in PD

Used primarily to treat chronic myeloid leukemia (CML), nilotinib hydrochloride is a drug that induces autophagy in misfolded proteins. Studies have hypothesized that low doses in the brain can safely target α-syn and Tau proteins and reduce their concentrations via the same method [7]. The concern, however, is the risk of myocardial infarction and sudden death caused by corrected QT (QTc) interval prolongation [16].

An investigative trial headed by Pagan et al. studied the potential efficacy of nilotinib on patients with PDD and dementia with Lewy bodies (DLBs). The trial consisted of 12 participants with either PDD or DLBs, with Parkinsonian Hoehn and Yahr stage 3-5, who were randomly placed into a 150 mg (n = 5) or 300 mg (n = 7) group, with participants respectively taking 150 mg and 300 mg of nilotinib once daily for six months, followed by a three-month follow-up. PD diagnoses were given by the UK Brain Bank Diagnostic Criteria, and MCI and severe dementia were assessed using MoCA within a range of 18-26. Participants were treated with PD medications, such as L-Dopa, for four weeks before study enrollment. Some patients who were treated with monoamine oxidase (MAO)-B inhibitors, selegiline or rasagiline, were also enrolled [7].

At two months after baseline, there was a slight decrease in CSF α-syn and a significant decrease in t-Tau for the 150-mg group. At six months, the 150-mg group exhibited increased CSF α-syn levels compared to the levels at two months, though it remained lower than at baseline. However, for the 300-mg group, there was no noticeable change in CSF α-syn over six months. CSF levels of t-Tau, however, decreased significantly after six months for the same group. Levels of plasma α-syn exhibited similar results to CSF α-syn in the 150-mg group; however, in the 300-mg group, levels decreased significantly at two months but returned to baseline at six months. Ultimately, the study suggests that nilotinib may have a stabilizing effect on α-syn levels in PD patients but also notes that a larger sample size, with a placebo group, is required to obtain more conclusive results [7].

In the second phase of his randomized control trial, Pagan et al. expanded on their previous one by including a placebo group and observing 75 participants, all of whom were diagnosed with PD as confirmed by the UK Brain Bank Diagnostic Criteria, Hoehn and Yahr stage 3-5, MoCA score 22 or higher, and UPDRS III motor score 20 to 40. The participants were randomly placed into three groups: placebo, 150 mg, and 300 mg, who, as the names suggest, were respectively given the sugar pill, 150 mg of nilotinib, and 300 mg of nilotinib once daily. These doses, administered over 12 months, were observed to have influenced CSF biomarkers such as dopamine turnover in the brain, oligomeric α-syn, and p-Tau. The research showed that the treatment was reasonably safe, though serious adverse effects (SAEs) appeared less frequently in the placebo group. There were also no differences between the rates of SAEs related to cardiovascular disease [16].

Lower doses (150-200 mg) of nilotinib have been shown to engage biomarkers of PD, such as α-syn levels and dopamine metabolism in CSF. Increased doses had varying effects. Over 12 months, CSF α-syn levels showed no change when treated with doses of 150 mg. However, oligomeric α-syn levels significantly decreased, possibly due to increased dopaminergic neuronal activity. Those treated with 300 mg did not exhibit these changes. There was also a significant decrease in CSF p-Tau, which agrees with previous trials conducted on animals [16].

In regard to motor and nonmotor outcomes, there were no impactful differences between the nilotinib groups and controls. The Parkinson Disease Questionnaire-39 and UPDRS showed little to no change; however, there was a slight change in MoCA scores that was not significant enough to be impactful in PD patients who already suffered from MCI. There was a more significant difference in UPDRS II and UPDRS III scores. At the 12-month mark, UPDRS II scores worsened for those treated with 300 mg of nilotinib. Likewise, the placebo group showed worsening scores 15 months after baseline. The 150-mg group did not show a significant change in this score; however, there was a 2.82 decrease in the UPDRS III score 15 months after baseline [16].

Wang et al., in their narrative review, observed that animal models and recent clinical trials have shown that antibodies targeting α-syn have been effective in immunotherapy, as α-syn contributes to the fibrillization of Tau and also forms the LBs in the brain characteristic of PD. Antibodies detect extracellular overexpressed α-syn, which are then marked for macrophages to eliminate. However, due to the multifaceted nature of α-syn, there is no panacea to target all posttranslational modifications, such as acetylation, phosphorylation, and truncation [8].

Immunotherapy can be categorized as active or passive. The former is characterized by introducing human-derived α-syn into the body, whereas the latter occurs when α-syn antibodies are injected, which triggers the body to form antigen-antibody complexes. Active immunization operates through the use of specific antigens to trigger an inflammatory response, making it the more viable option for patients with early PD and for populations at high risk, as opposed to passive immunization, which is ideal for mild and severe cases [8].

Due to the poor outcome of conventional PD drugs, interest in more targeted treatments has increased. Unfortunately, efforts to regulate α-syn production have, thus far, been restricted to laboratory trials. A novel approach to immunotherapy for α-syn control is research into nanoantibodies which, unlike current antibodies, are introduced intracellularly. A potential example is VH14, a micro-antibody that targets the non-amyloid component (NAC) region of α-syn. Brain shuttle modification or proteasome-targeting signal fusion can aid antibody-based drugs in entering the CNS [8].

## Conclusions

This systematic review investigated the role in the diagnosis and prognosis of PDD, while also touching on potential treatments. While there are many studies bolstering the use of α-syn and Tau proteins as biomarkers for PD and PDD, few are comprehensive enough to provide conclusive results. Typically, observational trials are relatively short, cohorts are relatively small and heterogeneous, and clinical procedures are not standardized - all of which are factors that potentially create inconsistencies in biomarkers and, in some cases, contradictory results. Studies have generally shown that concentrations of these proteins remain stable in the early phases of PD, which then increase as the disease progresses, implying that they reflect intensifying neurodegeneration over time. Addressing the shortcomings of the research may allow for a common consensus on the effectiveness of using α-syn and Tau in PDD diagnosis.
