# Correction: The Role of Alpha-Synuclein and Tubulin-Associated Unit (Tau) Proteins in the Diagnosis, Prognosis, and Treatment of Parkinson’s Disease: A Systematic Review

**DOI:** 10.7759/cureus.c253

**Published:** 2025-08-24

**Authors:** Yi Mon Lin, Devendar Banoth, Muhammad Hassaan Wali, Khava Bekova, Noor Abdulla, Simhachalam Gurugubelli, Safeera Khan

**Affiliations:** 1 Internal Medicine, California Institute of Behavioral Neurosciences and Psychology, Fairfield, USA; 2 Surgery, California Institute of Behavioral Neurosciences and Psychology, Fairfield, USA

This article has been corrected to address data discrepancies between the article text and PRISMA diagram. The following changes have been made:

**Abstract: **"**2,361 **participants" corrected to "**3,050** participants"

Results:

- **"194** failed quality appraisal" corrected to "**198** failed quality appraisal"
- "final list consists of **11** articles" corrected to "final list consists of **10** articles"
- PRISMA diagram: **"195** failed quality appraisal" corrected to "**198** failed quality appraisal"

